# Reducing disease and death from Artisanal and Small-Scale Mining (ASM) - the urgent need for responsible mining in the context of growing global demand for minerals and metals for climate change mitigation

**DOI:** 10.1186/s12940-022-00877-5

**Published:** 2022-08-26

**Authors:** Philip Landrigan, Stephan Bose-O’Reilly, Johanna Elbel, Gunnar Nordberg, Roberto Lucchini, Casey Bartrem, Philippe Grandjean, Donna Mergler, Dingani Moyo, Benoit Nemery, Margrit von Braun, Dennis Nowak, on behalf of the Collegium Ramazzini

**Affiliations:** 1 Program for Global Public Health and the Common Good, Global Observatory on Planetary Health, Boston College, Boston, USA; 2 Centre Scientifique de Monaco, Monaco City, Monaco; 3 Institute and Clinic for Occupational, Social and Environmental Medicine, University Hospital, LMU Munich, Munich, Germany; 4 Department of Public Health and Clinical Medicine, Umea University, Umea, Sweden; 5 Institute of Occupational Health, University of Brescia, Brescia, Italy; 6 TerraGraphics International Foundation, Moscow, ID 83843 USA; 7 Department of Environmental Health, Harvard T. H. Chan School of Public Health, Cambridge, MA USA; 8 Centre de recherche interdisciplinaire sur le bien-être, la santé, la société et l’environnement (Center for Interdisciplinary Research in Health, Wellbeing, Society and Environment, Université du Québec à Montréal, Montreal, QC Canada; 9 School of Public Health, Faculty of Health Sciences, Occupational Health Division, University of the Witwatersrand, Johannesburg, Republic of South Africa; 10 Department of Community Medicine, Faculty of Medicine and Health, National University of Science and Technology, Bulawayo, Zimbabwe; 11 Department of Community Medicine, Faculty of Medicine and Health, Midlands State University, Gweru, Zimbabwe; 12 Centre for Environment and Health, Department of Public Health and Primary Care, KU Leuven, Leuven, Belgium; 13 Environmental Science Program, University of Idaho, Moscow, ID 83843 USA

**Keywords:** ASM, Mining, Green energy transition, Occupational health, Environmental health, Global south

## Abstract

Artisanal and small-scale mining (ASM) takes place under extreme conditions with a lack of occupational health and safety. As the demand for metals is increasing due in part to their extensive use in ‘green technologies’ for climate change mitigation, the negative environmental and occupational consequences of mining practices are disproportionately felt in low- and middle-income countries. The Collegium Ramazzini statement on ASM presents updated information on its neglected health hazards that include multiple toxic hazards, most notably mercury, lead, cyanide, arsenic, cadmium, and cobalt, as well as physical hazards, most notably airborne dust and noise, and the high risk of infectious diseases. These hazards affect both miners and mining communities as working and living spaces are rarely separated. The impact on children and women is often severe, including hazardous exposures during the child-bearing age and pregnancies, and the risk of child labor. We suggest strategies for the mitigation of these hazards and classify those according to primordial, primary, secondary, and tertiary prevention. Further, we identify knowledge gaps and issue recommendations for international, national, and local governments, metal purchasers, and employers are given. With this statement, the Collegium Ramazzini calls for the extension of efforts to minimize all hazards that confront ASM miners and their families.

## Background

Artisanal Small-Scale Mining (ASM) is one of the world’s most dangerous occupations. The World Bank estimates that 100 million children, women and men work in ASM worldwide, mostly in remote rural areas of low-income and lower-middle-income countries. These miners often work under extreme conditions, the communities where they and their families live are heavily polluted, and ASM is responsible for high, but preventable rates of disease, injury, and premature death. ASM is increasing rapidly. Paradoxically, a key driver of this growth is climate change mitigation.

Climate change mitigation increases the demand for metals used for low-carbon-technologies, such as cobalt and lithium. The increased demand of such minerals will increase large-scale mining as well as ASM activities in the sector. Large quantities of global cobalt are mined in the DR Congo, where 20–30% originates from ASM. It is widely recognized that mineral demand will continue to increase in the coming decades.

The goals of this statement, which the Collegium Ramazzini issued during the United Nations COP 26 meeting on Climate Change in November 2021 are to:

- Provide updated information on the neglected health hazards of ASM and on strategies for mitigation of these hazards in the context of rapidly growing global demand for minerals and metals to meet the urgent need for climate change mitigation;
- Raise awareness of ASM hazards among policy-makers and the public; *and*
- Call for urgent interventions against the grave dangers of ASM by international organizations, governments, employers, and minerals and metals purchasers.

The Collegium Ramazzini notes the gross injustice of ASM. While most ASM takes place in the Global South, in the same countries already suffering the most serious consequences of climate change, most who benefit from ASM are in the Global North and thus have a shared responsibility to encourage their governments to contribute to reducing ASM hazards. We cannot achieve climate change mitigation through the use of “blood minerals”.

## Main issues

Artisanal and Small-Scale Mining (ASM) is highly dangerous work associated with multiple occupational and environmental hazards. In most mines little consideration is given to health and safety. Governmental oversight is rare, especially in areas where ASM is illegal. Severe injuries such as falls from heights, crush injuries from cave-ins, and lacerations and amputations from unguarded machinery are common. Because there is little separation between working and living areas in ASM, miners, their families, and residents in mining communities are at risk of exposure to hazards associated with mining for 24 h each day, every day, throughout the year, often under very primitive conditions.

**Table:** 

Definitions “Small-Scale Mining” is defined as mining conducted by small companies with limited financial resources and limited numbers of miners. These mines typically use some forms of technology – mainly low-end and inexpensive technologies. “Artisanal Mining” is defined as mining conducted by an individual miner and family members. It is smaller than small-scale mining, involves mainly manual labor, has no financial support, and is usually not formalized. Both Artisanal and Small-Scale Mining take place mainly in Low- and Lower-middle income countries.	

Artisanal and small-scale miners are exposed in their work to multiple toxic hazards, most notably mercury, lead, cyanide, arsenic, cadmium, and cobalt:

- Mercury exposure occurs mainly in gold mining, where milled ore is mixed with mercury to form an amalgam, and the amalgam is then vaporized and produces highly toxic mercury vapor. Mercury exposure also occurs in mercury mining. Extensive exposure to both metallic and organic mercury occurs in ASM. Along with coal combustion, ASM is one of the world’s two largest sources of mercury pollution. Elemental, organic and inorganic mercury are toxic substances, all to be found in ASM, causing severe damage to the neurological, renal, digestive and immunological system. Many miners show symptoms of a chronic inorganic mercury vapor intoxication, which can also be found in affected communities.
- Cyanide exposure is another very serious hazard of gold mining and occurs when cyanide is used as an alternative to mercury in the separation of gold from ore. Cyanide adversely affects respiratory and cardiovascular health and is known to adversely affect the central nervous system.
- Lead, arsenic, and cadmium exposure occurs in mines where lead, arsenic, cadmium, gold and other metals occur together in the mineral ore. Lead is a neurotoxic substance, negatively affecting the pre- and postnatal cognitive development. Lead is a human carcinogenic substance. Lead exposure in ASM causes severe clinical symptoms such as anemia, abdominal pains, seizures, encephalopathy up to increased mortality. Arsenic is a carcinogenic substance which causes dermatological, pulmonary, and cardiovascular diseases. Cadmium is a carcinogenic substance. Exposure to cadmium negatively affects the renal function, immune responses, cardiovascular and skeletal health.
- Cobalt exposure occurs in cobalt mining. Exposure to cobalt can cause negative effects to the pulmonary, hematological, endocrine, and nervous system.
- All those exposures have multiple adverse health outcomes, including serious social implications.

Artisanal and small-scale miners are occupationally exposed to physical hazards, most notably accidents, airborne dust, and noise:

- Accidents in ASM are the main hazards for miners’ health. Miners lack safety equipment and training. Many mines are extremely unsafe workplaces due to the fact that safety regulations are lacking, neither enforced nor implemented by governments. This results in a variety of occupational hazards that are not controlled, thereby placing the health and safety of miners at risk.
- Levels of silica-laden dust tend to be especially high in hard rock ASM mines, and silica exposure increases the risk of death from respiratory diseases including silicosis, tuberculosis, lung cancer, and COVID-19.
- Noise levels in artisanal and small-scale mines are typically far above acceptable levels due to the poorly regulated use of dynamite and heavy machinery. Sustained noise exposure can lead to hearing loss, as well as cognitive and behavioral disabilities.

Artisanal and small-scale miners are at high risk of infectious diseases:

- The COVID-19 pandemic affects disproportionately ASM miners and their communities because hand washing facilities, face masks and provisions for physical distancing are rarely available.
- Silica exposure, which is widespread in ASM, weakens the immune response thus increasing vulnerability to tuberculosis and COVID-19 infections.
- Rates of enteric diseases are high due to frequent lack of hygiene and sanitation facilities in the mines and insufficient access to clean water and food.
- Sexually transmitted diseases including HIV/Aids are common among mobile men with money (MMM), including miners.

Women and children in ASM and in ASM communities face unique and severe risks. Pre- and postnatal exposure to neuro-developmental toxins pose a specific risk for women at childbearing age and/or infants. Well known neuro-developmental toxins that are common in ASM are mercury, lead, and arsenic. Women may be subjected to sexual assault, violence, and psychological abuse, and they often face discriminatory work practices. Child workers are at risk of exploitation, physical and psychological abuse, and are subjected to working conditions where physical strain and chemical exposures may result in lifelong disabilities.

Artisanal and small-scale miners’ health and the health of their families are further eroded by corruption, malnutrition, violence, lack of access to health care and lack of education. Poverty is the main driving force for ASM, and its impacts are worsened by a lack of adequate and collaborative formalization efforts in the ASM sector.

Demand for metals: Strong and rising global demand for metals is the major driver of increases in ASM, and mineral demand is expected to continue to increase by as much as 450% until 2050. Climate change is a critical factor in this increased demand, because vast quantities of key minerals are needed for low-carbon energy technologies such as solar and wind power, e-vehicles, and new-generation batteries. The impacts of this increased demand are expected to be massive in countries such as the Democratic Republic of Congo, which holds roughly half of the world’s cobalt reserves (Appendix, Table 10). The rising price of metals, notably gold where the prices doubled in the last decade, will further fuel increases in ASM. Nevertheless, novel technologies to foster low-carbon technologies are on the rise, which may require a reduced amount of these critical minerals or use recycled material. The World Bank estimates the recycled content rate for cobalt at 32%. This proportion is projected to stay constant until 2050.

As climate change impacts become more severe, economic uncertainty increases, and metal prices remain high, more people in low-income and lower-middle-income countries will turn to ASM in search of livelihoods. Spikes in metal prices have been associated with large-scale environmental and occupational health tragedies in the past in Zamfara (Nigeria), Dakar (Senegal), and the DR Congo. In the absence of decisive action by governments and metal purchasers, these tragedies will multiply.

Knowledge gaps: The full number of artisanal and small-scale miners globally is not known and may be substantially greater than the current World Bank estimate of 100 million, given that a lot of ASM takes place in remote rural areas of Low-income and Lower-middle-income countries and is illegal in some places. For artisanal and small-scale gold mining the number of miners is estimated to be between 10 and 19 million. For all the different sectors of ASM accurate information on the number, gender distribution, and age distribution of artisanal and small-scale miners and on the numbers of people living in ASM communities in all countries are lacking but would be useful for planning health and social services.

Patterns of disease, injury and premature death in artisanal and small-scale miners are poorly defined. The contribution of ASM to the global burden of disease is inadequately charted. Consequently, the health impact of interventions in the field of ASM can also not be measured adequately.

Little is known about the local economic factors that impel populations to shift from subsistence agriculture to subsistence mining for their livelihood. ASM is a source of income diversification in many regions where farming is seasonal. In regions experiencing reduced crop yields as a result of climate-change-related alterations in weather patterns, it is possible that agricultural communities are already shifting to ASM for income stability. In Latin America, many native communities started to deforest and obtain minerals with local miners. However, it is controlled by the community. Better understanding of these relationships is needed, especially in supporting local development on top of governmental actions to improve climate adaptation strategies.

Artisanal and small-scale gold mining are the world’s largest sources of anthropogenic mercury pollution. UNEP’s “Minamata Convention on Mercury” supports programs to reduce and replace mercury in Artisanal and Small-scale Gold Mining (ASGM). The supply of mercury for ASGM areas is widely uncontrolled, including illegal trade and informal mercury mining. More information is needed on the production, supply, and market for mercury used in gold extraction (Appendix, Table 11).

Generations of children in ASM villages are exposed prenatally to mercury, lead, arsenic, cadmium, cobalt, manganese or other toxic pollutants generated by mining. Children ingest these toxic materials in breast milk; they play and grow up in polluted, dusty areas contaminated by metals and other hazards; and they start to work as miners even before they reach puberty. The lifelong health consequences of those exposures are very different from the health effects for healthy adult workers. Because they eat more food, drink more water, and breathe more air per Kg body weight compared to adults, children are disproportionately heavily exposed to hazardous materials. Too little is known about the pre- and postnatal health hazards for occupationally exposed children. Clinical and epidemiological studies of children in ASM communities are urgently needed.

## Conclusion/ recommendations

The Collegium Ramazzini calls urgently for extended efforts to minimize all hazards related to ASM. International organizations, governments at all levels – national, state or provincial, and local - and all employers - large and small, public and private - must fulfill their responsibilities to protect the health of all workers in ASM and to create occupational health and safety programs that will reduce risks of disease, injury and premature death among artisanal and small-scale miners. This call becomes particularly urgent in the context of growing global demand for minerals and metals for climate change mitigation. The Collegium Ramazzini urges non-governmental organizations to accept the challenge of reducing the grave hazards that confront artisanal and small-scale miners and their families.

Responsibilities of the International Community: As the world increases its reliance on renewable energy and demand grows accordingly for minerals and metals, the Collegium Ramazzini calls upon UN agencies and the international community to give special attention to the issue of ASM and to urge actors in the global supply chain to adopt codes of conduct that document and declare that all metals in commerce have been extracted under conditions that assure safety and health. Specifically:

- We urge the United Nations to adopt a Convention on the Safety and Health of ASM, in which member nations commit to establishing both domestic and international protections against the abuse of ASM workers and their families.
- We urge WHO and ILO to launch an international movement focused on quantifying and reducing the health hazards that arise from ASM.
- The World Bank and all other intergovernmental organizations engaged in facilitating global trade, including the International Trade Organization and the Organization for Economic Cooperation and Development, should continue to support countries managing their resources and promote decent and ethical supply chains.

Responsibilities of Governments

- It is imperative that governments put systems and processes for safeguarding the health and safety of artisanal and small-scale miners in place. Chief among these is the need to improve the access to occupational health and safety services.
- Governments should develop transparent ASM management systems that include meaningful participation from all relevant stakeholders.
- Governments should decriminalize ASM in areas where it is illegal and forge collaborative partnerships to improve access to health services. They should support communities in developing a formalization framework that is protective of human and ecological health, and that protects both vulnerable groups and sensitive ecosystems.
- Governments should adapt and enforce international conventions such as the Basel Convention, the Stockholm Convention, and the Minamata Convention on Mercury.
- Governments should adopt and enforce all the ILO conventions and recommendations for health and safety in the mining industry such as the Safety and Health in Mines Convention (ILO No. 176), the Minimum Age Convention (ILO No. 138), or the Worst Forms of Child Labour Convention (ILO No. 182), the OECD’s Due Diligence Guidelines and develop and adopt legislation obliging enterprises to conduct environmental and human rights due diligence in cooperation with all stakeholders involved and affected.
- Governments should adopt the basic occupational health services model that seeks to integrate occupational health to primary health services.
- Governments should provide ASM communities with tools and resources to improve occupational health and safety, community health, environmental sustainability, and remediation needs.
- Governments should establish systems that will enable miners to readily access markets for mercury-free, environmentally, and socially responsible mineral extraction.

Responsibilities of Employers

- Employers have legal and moral responsibilities in all countries to provide a safe and healthy working environment to all miners in their employ.
- Multinational companies must apply the same occupational health and safety standards and environmental standards in countries where they operate – in High-income countries as well as in Low- and Lower-middle-income countries.
- Employers need to provide adequate access to remedy for victims of rights abuses and provide grievance mechanisms.

Responsibilities of Mineral Purchasers

- Mineral purchasers have the responsibility to perform due diligence upstream the supply chain of minerals by diligently investigating the supply chain of minerals they buy. It is unacceptable to purchase minerals produced in environmentally or socially harmful conditions, and it is inadequate to defer accountability due to a lack of knowledge.
- Policies for responsible mineral supply chains must be developed and followed, including traceability of minerals, refusal to do business with suppliers who cannot meet responsible production criteria, and consistent monitoring of suppliers.
- Purchasers should publicly report all measures and due diligence steps, including their risk reduction strategies, their risk management plan, and their monitoring efforts. In line with OECD Due Diligence Guidance for Responsible Supply Chains of Minerals from Conflict-Affected and High-Risk Areas, companies should include those findings in their annual reports.
- Purchasers should also disclose mineral supply information to consumers, including any knowledge or lack thereof of production or trade conditions, criteria met for sustainable and responsible ASM practices, and supply chain information about the minerals used in the product.

Further elaboration of these recommendations is provided in the attached Appendix and its Annexes.

## Figures and Tables

**Fig. 1:**
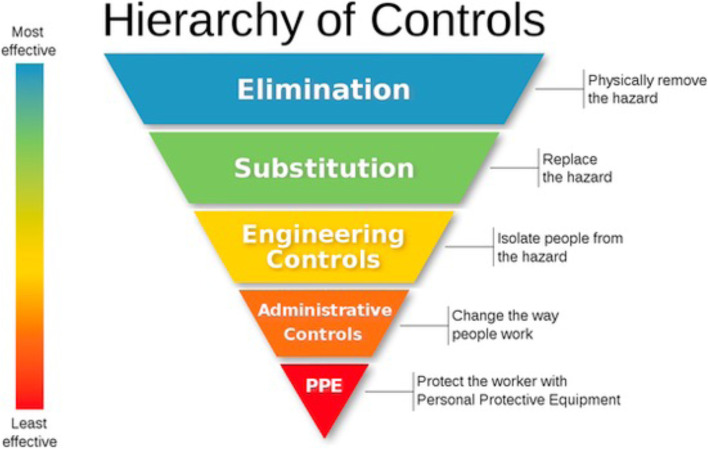
National Institute for Occupational Safety and Health (NIOSH). Hierarchy of controls [Internet]. Centers for Disease Control and Prevention. Centers for Disease Control and Prevention; 2015. Available from: https://www.cdc.gov/niosh/topics/hierarchy/default.html

## Data Availability

Not applicable.

## Appendix

This review gives relevant details and elaboration on hazards confronting miners and mining communities. It is structured in eight boxes, each box containing information on a subsection of risks.

- Table 1 presents a hierarchy of occupational health and safety standards for miners.
- Table 2 describes the physical hazards and injuries of ASM.
- Table 3 list the toxic hazards of ASM.
- Tables 4, 5 and 6 describes the dust (Table 4), infectious diseases (Table 5), and noise (Table 6) hazards of ASM.
- Tables 7 and 8 describes the psychosocial hazards of ASM (Table 7) and the particular hazards confronting women and children (Table 8).
- Table 9 elaborates on the impact of climate change on ASM and on the impact of ASM on the environment.
- Table 10 describes the rising demand for metals for climate change mitigation.
- Table 11 discusses gaps in research and the need for more data on ASM and its hazards.

**Table 1:** Occupational Health and Safety: Strategies for prevention of exposure to toxic materials and hazardous conditions in ASM are classified as primordial, primary, secondary, and tertiary

**Primordial prevention:** Actions designed to prevent dangerous exposures from ever occurring. *Examples:* Removal of mercury from Artisanal and Small-Scale Gold Mining. **Primary prevention: **Strategies for preventing disease by reducing exposures. *Examples:* Controlling workplace exposures to metals through process enclosure, exhaust ventilation, administrative controls, and personal protective equipment. While important, these measures are less effective than outright bans or substitution with less hazardous materials. Their application is guided by the Hierarchy of Controls framework (see below). Standard-setting is an important aspect of primary prevention of exposure to toxic metals. In most countries, standard-setting is a legal as well as a scientific process and is often guided by the paradigm of Risk Assessment and Risk Management. Risk assessment/risk management is sometimes modified by application of the Precautionary Principle [1–3]. **Secondary prevention:** Methods for early detection of disease before the appearance of symptoms, complications, or spread, through biological monitoring and health surveillance. *Example*: Blood lead and urinary mercury screening to detect exposures leading to biochemical changes related to minimal or slight symptoms [4, 5]. Biochemical analyses of precursors of symptoms or slight symptoms, e.g. erythrocyte zinc protoporphyrin in occupational lead exposure [5]. **Tertiary prevention:** Methods to prevent severe consequences of disease, such as disability or death. *Examples: *Chelation therapy of acute, high-dose exposures to metals [6]. Application of this classification of preventive strategies to the prevention of occupational exposures in ASM is guided by the “*Hierarchy of Controls*” framework, developed by the US National Institute for Occupational Safety and Health. Figure 1: National Institute for Occupational Safety and Health (NIOSH). Hierarchy of controls [Internet]. Centers for Disease Control and Prevention. Centers for Disease Control and Prevention; 2015. Available from: https://www.cdc.gov/niosh/topics/hierarchy/default.html Minimizing or eliminating exposures at the source before exposure ever occurs—*primordial prevention*—is the single most effective and cost-effective means of preventing hazardous exposures. It is therefore listed first in the hierarchy of controls. Personal protective equipment (PPE), while very important, is the least effective of these control strategies and thus is listed last. Insufficient training and education is a pervasive problem in ASM. Miners are often unaware of the hazards, which are largely shaped by the social and communal setting and influenced by informal or illegal working situations and a lack of OHS management organizations. Evaluation of the few OHS programs in ASM has not been undertaken but would be crucial. Long-term consequences of hazard exposure are not researched sufficiently and analogous legislation and regulation in the field of ASM lack attention and are low on the political agenda [7–9].	

**Table 2:** Physical Hazards and Injuries

Lack of safety in mining processes is the main hazard for the miners’ health [10]. Artisanal and small-scale mines are poorly mechanized and use rudimentary mining methods thereby exposing themselves to a wide variety of occupational hazards. Artisanal and small scale miners lack expertise and competency in conducting workplace risk assessments due to a lack of training and education [11, 12]. This results in a multiplicity of occupational hazards that are not controlled thereby placing the health and safety of miners at risk. Poor mechanization in artisanal and small scale mines is often associated with unsafe working processes. The miners also lack knowledge and competence in the application of the hierarchy of controls leading to unsafe workplaces [13]. Falls from heights, mine collapses and crush injuries are common challenges within this population [10, 14–17]. Miners are faced with extreme working conditions on a daily basis. The non-ventilated, small, and unsecured tunnels can fully or partly collapse and injure or kill workers. Blasting of tunnels with insecure explosives, or a misapplication of explosives frequently harms miners. Especially open pit mines are unstable and collapse frequently and underground water mining is considered exceptionally hazardous. Miners are often unaware of risks because training and education is absent or insufficient [11]. The risks result in high fatality and injury rates, including “burns, eye injuries, fractures, impalement, and in some instances physical dismemberment” [10].	

**Table 3:** Toxic Hazards

Various toxicological hazards occur in different types of mining. 50–100 million women, infants, children and men in ASM settings are exposed to *mercury* [18, 19]. Concentrations of mercury in biological matrices of individuals living in mining settings are measured to be at toxic levels [20, 21]. Processing gold after extraction entails the smelting of amalgams, hence, research has shown that especially in artisanal and small-scale *gold* mining exposure to mercury is high among miners and communities [22, 23]. Thereby, highly toxic elementary mercury vapor is inhaled [19]. Consequently, several adverse health effects, especially neurological effects are observed [24, 25]. An estimated 25 to 33% of ASGM miners show the symptoms of chronic mercury vapor intoxication, meaning 3.3–6.5 million miners globally [20]. In ASGM 10 to 19 million miners are exposed to mercury [26]. The WHO identifies elemental mercury as hazardous to the nervous system [27]. In particular mercury vapor, as seen in ASGM, is also harmful to the kidney, the digestive and the immune system, potentially causing fatal organ failures. Additionally, behavioral and neurological effects are described after any mode of exposure to mercury [4]. The WHO [10] points out that *lead* poisoning in mining areas increases mortality and morbidity rates. Varying levels of lead, determined by geological factors, can be found in former and current ASM settings. In Kabwe/Zambia a former zinc-lead mine is a constant source of lead exposure for the children playing and living nearby the old tailing hill, thousands of children have very high lead levels in their blood, so the exposure from the uncovered tailing hill and the lead containing soil in the settlements have to be stopped urgently [28–31]. Scavengers are still exploring the old mining side and expose themselves as well [28]. Since 2010, several authors describe that children in a gold mine in Nigeria are highly intoxicated; approximately 400 fatalities due to lead poisoning among those children were reported [32–38]. Exposure to lead at many toxic sites in Low- and Lower-middle-income countries is a well-known risk factor for the cognitive development during pregnancy [39], an “estimated 820,000 women of childbearing age are at risk for lead exposure at these sites” [40]. Lead is an IARC 2A carcinogen with strengthening evidence more recently [41]. *Arsenic* is released from mining and processing. Arsenic is an IARC 1 carcinogen depending on the route of exposure and species associated with skin, bladder, lung, kidney, and liver cancer [42]. Arsenic can adversely affect adults and children. Depending on exposure levels and circumstances, various health consequences are observed, such as skin rashes and pulmonary and cardiovascular diseases. Children and infants exposed to arsenic frequently develop neuro-developmental and -behavioral disorders [43, 44]. *Cadmium* is a by-product of mining. Cadmium is an IARC 1 carcinogen, mainly associated with lung cancer, but also with cancer of the kidney and the prostate [45]. Additionally cadmium deteriorates the renal function, immune responses, cardiovascular and skeletal health [46]. The demand for *cobalt* sharply increases due to technological developments. Batteries used in novel electric vehicles and other technologies require cobalt as an essential resource. Mines in the Katanga Copperbelt in the Democratic Republic of Congo (DRC) are producing 60% of the metal used worldwide [47]. 15–30% are estimated to originate from ASM [47–49]. Environmental pollution in mining areas and lack of separation of living and working spaces, sets not only miners themselves but entire communities at risk of experiencing adverse health effects. Cobalt exposure was found to be correlated with dust exposure causing long-term damage of inter alia, the cardiovascular- and pulmonary system [50]. Additionally, DNA damage of children living in mining areas in the DRC was found, indicated by high levels of 8OHdG in urine biomonitoring [47]. Indication exists that birth defects of children are related to *cobalt* and *copper* mining [51]. Increased sustainability of cobalt mining in terms of environment and health is urgently needed to protect the most vulnerable. Not counting as a so-called *blood metal*, which are related to high rates of conflict and violence, cobalt nowadays remains unregulated. Unlivable and hazardous conditions cannot continue to affect an increasing number of individuals who are supplying minerals which are classified as essential to sustain western economies [47]. *Cyanide is* used as an alternative to mercury in ASGM and adversely affects respiratory and cardiovascular health and is known to adversely affect the central nervous system [52]. Specific data and knowledge about the health risks related to cyanide exposure in mining settings is lacking [53].	

**Table 4:** Dust Related Diseases

Respiratory diseases are worsened and caused by *silica containing dust* exposure in ASM settings. Workers and mining communities are at risk of developing silicosis, which can lead to decrease of pulmonary function and increases the risk of (silico-)tuberculosis and lung cancer. Often data collected in mining areas does not reflect the real burden of disease related to dust exposure because consequences might appear delayed once miners have already left the workforce. Sick individuals often also move from mining communities when they fall ill and are thus frequently excluded from data describing the incidence of disease. Predispositions and positive correlations between dust exposure and incidence of cancer in mining communities, as well as infectious diseases, such as tuberculosis and HIV, are also described [46, 54–56]. A combination of HIV infection and silicosis has a more than additive risk of TB infection in excess of fifteen-fold [57, 58]. This is very important considering that artisanal and small-scale miners have a high burden of HIV and silicosis. It is of paramount significance for governments to put systems in place to improve access to health services for artisanal and small-scale miners [57, 58].	

**Table 5:** Infectious Diseases

The spread of *infectious diseases* in artisanal and small scale miners is facilitated due to lacking *hygiene* and *sanitation* facilities and insufficient water and food safety. This concerns not only miners, but also their families, especially children and women. In combination with dust, described above, and a high prevalence of silicosis, respiratory diseases such as COVID 19 and tuberculosis are extremely hazardous. The SARS-CoV-2 pandemic disproportionately affects miners and mining communities [59]. Prevention efforts, such as hand washing facilities and face masks are often not available in mining communities. Production of minerals in ASM mostly continued, however at a lower rate. Lower demand from the international community led to lower prices and the international measures implemented to control COVID-19 affect trade and, therefore, the socioeconomic conditions of miners. Prices of gold dropped by about 20%, diamonds and tanzanite prices by 60–70% [60]. The living and working conditions have, hence, worsened. Children were observed more frequently at mining sites because schools were closed. The supply of essentials, such as food and water, was disrupted in some places. Efforts of the governments to stop the virus from spreading shifted attention away from long-term programs, such as conflict prevention and peace-making efforts and the socioeconomic circumstances led to higher crime and robbery rates in some communities. In July 2020 international trade recovered to some extent and prices rose. The return to normal production, however, goes hand in hand with low availability of and compliance to COVID-19 prevention efforts, posing an additional risk to the vulnerable communities [60, 61].	

**Table 6:** Noise

Mining is characterized by high *noise* exposures which can lead to permanent noise induced hearing loss. In ASM, the lack of personal protection equipment (PPE) and safety and health preventive mechanisms presents a significant challenge and is responsible for high cases of noise induced hearing loss [62]. Basu et al. (2015) portrayed the setting in ASGM in Ghana, identifying high *noise* exposure in various steps of the mining process [46]. Inter alia dynamite usage and grinding with generator powered machines were described as such exposures. According to the WHO, levels of noise in ASM can exceed acceptable levels for preventing hearing loss [10]. Additionally, cardiovascular, as well as cognitive and behavioral disabilities, are correlated to noise exposure in ASM.	

**Table 7:** Psychosocial Hazards

In addition to biological hazards the miners’ health is deteriorated by psychosocial hazards [46]. The quality of life is negatively affected [46, 63]. It is of great importance to recognize that those benefiting from mining are situated in the Global North and consistently externalize environmental and health risks and costs to countries in the Global South intensifying these psychosocial hazards. A variety of biopsychosocial hazards increase morbidity and mortality rates among miners. A lack of Occupational Health and Safety (OHS) regulations worsens the exposure and the consequences of exposure [64]. Psychosocial hazards include a decline in biodiversity and a displacement of indigenous communities. Prostitution, criminal activities, violence, and substance abuse are frequently observed in the mining setting. The lack of healthcare facilities and efforts to formalize health insurance and social security mechanisms, as well as the present informal or illegal employment situations contribute to hazardous living and working conditions. Women, in the role of workers, caretakers and mothers, are exposed to a variety of risks in mining settings. Child labor is a main risk factor for children living in mining communities [65, 66]. Miners, their families and the communities they live in, are exposed to prostitution, violence, criminal activities and substance abuse [46]. *Coltan* mines, almost exclusively found in the east of the DRC, are often at the center of violent conflicts; militia groups control these territories exploiting workers. These *blood minerals* are mostly exported, highlighting a responsibility of the internationals to ensure sustainable, transparent and non-violent extraction of coltan [67]. The requirement to only export resources labeled ‘*conflict-free’* to counteract violence and war was implemented for Congolese minerals. This ban, however, is criticized sharply for lowering the income and, thereby, worsening the conditions of miners in geographically remote areas, where control and, therefore, labeling is impossible. Secondly, strain is put on public authorities in a politically unstable setting where powers and legitimacy are not easily defined and production remains informal to a great majority [68]. *Tin, tantalum,* and *tungsten* (3 T minerals), as well as other minerals, are still part of the armed conflict in the DRC today. Notably, however, these are not a cause but a symptom of instability, conflict and poverty [69]. These arguments strongly relate to current formalization efforts. Top-down formalization efforts, in an environment where most employment is informal, frequently worsen living and working conditions of employees. Livelihoods that depend on the income from ASM are put under pressure by an increase of legitimacy to cooperate mines. A combination of bottom-up and top-down efforts has to be found [68]. A lack of provision, financing and regulation of healthcare services leads to insufficient availability of healthcare facilities and, due to the absence of insurance schemes, to non-affordable services. Nevertheless, this relates to the informality of most of the mining sector: Not only are health insurance schemes missing, but the lack of formal employment contracts, and financial and social protection also pose a great uncertainty to individuals, with an ever-present fear of catastrophic losses [65, 66, 70].	

**Table 8:** Hazards Confronting Women and Children

Women are often affected disproportionately. Estimates suggest up to 50% of miners are female in some African artisanal and small scale mines [71]. Because they are mainly involved in processing or transporting ore, they may not be recognized as miners or included in the statistics [72]. Often mining is experienced as a women’s sole way of gaining financial independence. It is reported that women engage in informal and illegal activities more frequently than men [70]. As workers in mines, or as those responsible for processing the ore, women are exposed to all hazards described above. Women’s reproductive health suffers and maternal health decreases. In ASGM, smelting is commonly seen as the women’s task and results in exposure to mercury fumes. This exposure can span from prenatal to adulthood, with adverse neurological effects on whole generations in working as artisanal and small scale miners [73]. Additionally, women are at a high risk due to isolation in mining settings and physical and sexual abuse. Sexual transmitted diseases (STDs) are frequently reported among women. As a result of structural gender inequalities women benefit from even fewer OHS services than men [10, 11, 64, 74]. Child labor in ASM is common and requires specific attention. According to the ILO [75] about one million children worldwide are working in mines. Sometimes up to half of the miners are below the age of 15. These children work in “life-threatening conditions, subject to violence, extortion and intimidation” [75], not able to seize some of their fundamental human rights. Since life chances are largely defined by early life years, growing up in mining communities and later working in mines diminishes an individual’s potential drastically. Firstly, weak maternal health determines an infant’s start to life and, thereby, his or her later health state. Children laborers have reduced access to education and, hence, few chances to escape from the hazardous settings. The biological hazards described above present a particular risk to the still developing children. Mercury can affect fetuses and children extensively and adverse health effects are reported. Malnutrition and adverse musculoskeletal consequences are described to occur among child miners [71, 76, 77]. Because of the high mobility of ASM communities, the presence of many young men who are without their families, the daily flow of cash, and the high rates of alcohol and substance abuse, prostitution is part and parcel of mining life [46, 78]. Like their mothers, girls are exposed to sexual and gender-based violence and exploitation, often resulting in pregnancies and STDs. Boys are not excluded from forced child prostitution [79]. In addition, substance abuse is reported among child miners. Financial incentives and peer pressure are usually the reasons children engage in mining activities [11, 65, 66, 70].	

**Table 9:** Environmental Impacts and Climate Change

All stages of ASM negatively impact the environment, contribute to climate change and are responsible for declines in biodiversity. Landscape destruction occurs when exploring, exploiting and closing mines. Furthermore, toxicological and waste pollution of waters, soil, and air is related to the processing of minerals and inappropriate disposal. Due to a lack of training and education, and formalization and control in the sector, these environmental damages continue to worsen the situation in mining settings. Indigenous communities are increasingly displaced, cultural deprivation accompanies the environmental devastation, therefore, suffering the long-term consequences of the environmental damage [11, 80–83]. These environmental impacts urgently need increased attention.	

**Table 10:** Rising Global Demand for Metals

Climate change mitigation increases the demand for metals used for low-carbon-technologies such as aluminum, chromium, cobalt, copper, graphite, indium, iron, lead, lithium, manganese, molybdenum, neodymium, nickel, silver, titanium, vanadium and zinc [84]. Solar cells, wind turbines, high-efficiency storage batteries, and electric vehicles are currently essential for the transition to a low-carbon economy [85]. The larger part of the minerals is mined using conventional methods, nevertheless the increased demand of such minerals will increase the ASM activities in the sector likewise, as, for example cobalt [86]. The World Bank projects that renewable energy systems will require significantly more minerals and metals than current fossil-fuel-based energy supply systems and that global demand for minerals and metals will continue to increase for many decades [49, 84]. One critical factor in this increase is the impact of climate change. By 2050, mineral production will need to meet rapidly growing demand for low-carbon energy technology such as solar and wind power, e-vehicles, and the batteries needed to store energy for “green” energy alternatives [49]: “Under a 2-degree scenario production of graphite, lithium, and cobalt will need to be significantly ramped up by more than 450% by 2050—from 2018 levels—to meet demand from energy storage technologies” Few can predict the impact of a 450% increase in cobalt demand on the Democratic Republic of Congo, which holds roughly half of the world’s cobalt reserves, and where 15–30% is produced via ASM [47–49]. Nevertheless, novel technologies to foster low-carbon technologies are on the rise, which may require a reduced amount of these critical minerals or use recycled material. The World Bank estimates the recycled content rate for cobalt at 32%. This proportion is projected to stay constant until 2050 [49].	

**Table 11:** Identification of Data Gaps and Lack of Knowledge

It is clear that low-carbon technologies are mineral intensive and will require large increases in global metal production. What isn’t clear is how climate-impacted populations shift from subsistence agriculture to subsistence mining. ASM is a source of income diversification in many regions where farming is seasonal; in regions experiencing reduced crop yields as a result of altered weather patterns, it is possible that agricultural communities are already shifting to ASM for income stability [82, 87–90]. Better understanding of this relationship is needed, especially in supporting local development of sustainable climate adaptation strategies. ASGM is the largest source of global anthropogenic mercury release [91], but relatively little is known about where the mercury is sourced and traded [92]. With the support of UNEP’s “Minamata Convention on Mercury” more data will become available and programs such as PlanetGOLD will help to address the issue of reducing and replacing mercury in ASGM [93]. The supply with mercury for ASGM areas is widely uncontrolled, including illegal trade and informal mercury mining [92]. More information is needed on the production, supply, and market for mercury used in gold extraction.	

## Abbreviations

ASGM: Artisanal and Small-scale Gold Mining
ASM: Artisanal and Small-scale Mining
COP26: 26th UN Climate Change Conference in Glasgow
COVID-19: Coronavirus disease 2019
HIV: Human Immunodeficiency Virus
ILO: International Labour Organization
MMM: Mobile Men with Money
OECD: Organization for Economic Collaboration and Development
PPE: Personal Protection Equipment
UN: United Nations
WHO: World Health Organization
